# Exosomal miRNAs in prenatal diagnosis: Recent advances

**DOI:** 10.1097/MD.0000000000038717

**Published:** 2024-07-12

**Authors:** Keqin Jin, Shuangshuang Shen, Ruyong Shi, Xiayuan Xu, Min Hu

**Affiliations:** a Genetic Laboratory, Jinhua Maternal and Child Health Care Hospital, Jinhua, China; b Prenatal Diagnostic Center, Jinhua Maternal and Child Health Care Hospital, Jinhua, China; c Department of Ultrasound Medicine, Jinhua Maternal and Child Health Care Hospital, Jinhua, China; d Gynaecology and Obstetrics, Jinhua Maternal and Child Health Care Hospital, Jinhua, China.

**Keywords:** exosome, miRNA, prenatal diagnosis

## Abstract

Exosomes, small membranous microvesicles released by cells, contain a range of bioactive molecules, including proteins and miRNAs, which play critical roles in intercellular communication and physiological and pathological processes. Current research suggests that exosomal miRNAs could serve as valuable biomarkers for prenatal diseases, offering a noninvasive method for early detection and monitoring. Studies linking exosomal miRNAs to various birth defects, including fetal growth restriction, urinary tract malformations, cardiovascular system malformations, and hereditary diseases like Down syndrome, were discussed. However, there are some conflicting study findings due to different exosome separation methods. Here, we also discussed exosome separation methods, emphasizing the importance of method selection based on specific purposes and sample types. Further studies are needed to standardize isolation techniques, understand the specific mechanisms underlying exosomal miRNA function, and develop reliable noninvasive prenatal diagnostic indicators. Overall, exosomal miRNAs show promise as potential biomarkers for prenatal diagnosis, but further research is necessary to validate their clinical utility.

## 1. Introduction

Exosomes, membranous microvesicles secreted by living cells, encapsulate molecules such as proteins, messenger RNAs (mRNAs), and microRNAs (miRNAs).^[1]^ These exosomes modulate various physiological and pathological functions in recipient cells, participating in a broad range of biological processes. The miRNAs contained within exosomes disseminate biological information through intercellular communication, influencing physiological and pathological processes such as tumor growth and development, inflammatory response, immune reaction, blood coagulation, and the repair of damaged cells.^[2]^ Given their pivotal role, exosomal miRNA holds significant potential in disease diagnosis, therapeutic strategies, and disease prevention.^[3–8]^ Prenatal screening and diagnostics are crucial for mitigating the occurrence of birth defects, and noninvasive early detection methods have garnered substantial academic attention.^[9]^ In recent years, the potential clinical value of exosomal miRNAs in prenatal diagnostics has attracted increasing interest from researchers.^[10]^ The integration of exosomal miRNAs analysis into prenatal diagnostics represents a promising advancement in noninvasive prenatal testing (NIPT). Moreover, the noninvasive nature of this approach minimizes risks to both the mother and the fetus. As research progresses, the standardization of exosomal miRNA isolation and analysis techniques will be crucial for their widespread clinical application. The future of prenatal diagnostics could be significantly enhanced by the adoption of these advanced molecular tools, ultimately leading to improved maternal and fetal health outcomes. Here, this article presents a comprehensive overview of the application prospects of exosomal miRNAs in antepartum diagnosis.

## 2. Exosomes and exosomal miRNAs

Exosomes, nano-scale vesicles derived from the cell membrane, are roughly 30 to 150 nm in diameter. Remarkably, virtually all types of viable cells can release exosomes when multiple vesicles fuse with a cell membrane. These exosomes encapsulate a plethora of bioactive molecules such as proteins, lipids, messenger RNAs (mRNAs), and microRNAs (miRNAs), exhibiting significant correlations with diverse physiological processes, including intercellular communication, cancer metastasis, immunomodulatory activities, and the transmission of infectious agents. Among these, miRNAs, an endogenous class of small, non-coding single-stranded RNAs spanning 18 to 25 nucleotides in length, play a pivotal role in post-transcriptional regulation of gene expression. Luo et al^[11]^ demonstrated that placental-specific miRNAs derived from human chorionic villus trophoblast cells could enter the maternal blood circulation via exosomes. The research by Cuffe et al^[12]^ substantiates that during pregnancy, exosomes originating from the placenta are released into the peripheral blood circulation. The increased abundance of miRNA molecules in the peripheral blood of patients with certain pregnancy complications suggests that disorders during pregnancy and the evaluation of fetal development can be predicted via miRNA molecules within these exosomes. In addition, given the relative stability of exosomal miRNAs in blood circulation^[13]^ and the ability of exosomes to directly or indirectly influence the entire course of pregnancy,^[14–17]^ they could potentially serve as a novel methodology for disease prediction and diagnosis.

Overall, the integration of exosomal miRNA analysis into prenatal diagnostics represents a promising advancement in noninvasive prenatal testing (NIPT). This noninvasive approach reduces the risks associated with traditional invasive methods such as amniocentesis and chorionic villus sampling, thereby providing a safer alternative for both the mother and the fetus. Additionally, the use of exosomal miRNAs could facilitate earlier interventions and better monitoring of pregnancy health, ultimately improving maternal and fetal outcomes. As research in this field advances, it will be crucial to standardize the techniques for isolating and analyzing exosomal miRNAs to ensure their reliable and widespread clinical application. The future of prenatal diagnostics could be significantly enhanced by adopting these advanced molecular tools, leading to more effective and personalized maternal-fetal healthcare.

## 3. Exosomes separation

Exosomes are currently isolated using a wide range of technologies, including centrifugation, precipitation, size exclusion, immunoaffinity, microfluidic technology, and magnetic separation techniques based on aptamers. However, the method has distinct advantages and disadvantages (Table 1). Centrifugation methods, including differential centrifugation and density gradient centrifugation, are traditionally used to isolate exosomes from various sample sources.^[18]^ However, they present high equipment costs and extended operation times. Density gradient centrifugation efficiently purifies exosomes, particularly for functional evaluations,^[19]^ but its complexity restricts widespread use. Precipitation strategies including polymer precipitation and protein organic solvent precipitation, offer simplicity and cost-effectiveness. However, they may yield sub-optimal purity, especially in removing impurities like lipoproteins and PEG chemicals during polymer precipitation. This method is primarily suitable for RNA analysis.^[20]^ polymer precipitation and protein organic solvent precipitation’s performance can also be influenced by factors like temperature, pH, and ionic strength. Particle size resolution methods include ultrafiltration and size-exclusion chromatography. Ultrafiltration offers convenience, time efficiency, and affordability, suitable for diverse sample types like cell culture supernatants, urine, pleural fluid, and abdominal fluid, albeit with a limited purity. Size-exclusion chromatography is suitable for extracting exosomes from small-volume (microliters and milliliters) samples like plasma, serum, and saliva, primarily used in functional studies and biomarker detection within these samples. The immunoaffinity method is beneficial for exosome marker detection and clinical diagnostic research, but its high reagent cost may hinder large-scale research. The microfluidic technique, a promising methodology, provides automation benefits for exosome isolation and detection, particularly advantageous in clinical diagnosis. However, its separation efficiency depends on apparatus quality, and validation of selectivity and specificity is crucial. Aptamer-based magnetic separation is ideal for exploring exosome functions and biomarkers in clinical diagnosis and prognostics. However, its limited aptamer repertoire restricts its application.

**Table 1:** Contrasting advantages and disadvantages of exosomes isolation techniques.

Isolation technique	Potential advantage	Potential disadvantage
Differential centrifugation	For functional and biomarker analysis of exosomes from varied sample types.	Expensive equipment, prolonged operation.
Density gradient centrifugation	Efficiently isolates high purity exosomes for functional studies, biomarker detection, and content analysis from cell culture supernatant or tissue samples.	Complex operation and Not suitable for small sample sizes.
Polymer precipitation	Simple operation, suitable for RNA analysis of the sample.	Low yield, poor purity, and difficult to remove lipoproteins, PEG and other chemicals such as magazines, affecting the bioactivity of exosomes
Organic solvent precipitation	Simple, rapid and cheap Suitable for detection of diverse biomarkers in various clinical patients.	Subject to various factors, such as temperature, pH and ionic strength.
Ultrafiltration method	Simple operation, time saving, low cost, high output and efficiency. Utilized for extracting exosomes from diverse sample matrices, including cell culture supernatant, urine, pleural fluid and ascitic fluid, for diverse clinical/activity research endeavors.	Insufficient purity
Size-exclusion chromatography	It is suitable for the isolation of exosomes from plasma, serum and saliva (microliter and milliliter) for functional study, marker detection and content analysis	Time-consuming
Immunoaffinity method	It is suitable for the study of marker detection and clinical diagnosis of exosomes	Reagents are expensive and not suitable for large samples.
Based on microfluidic method	It can be automated and has great advantages in the study of exosome biological function and clinical diagnosis of diseases	The separation efficiency depends on the device, and its selectivity and specificity remain to be verified
Magnetic separation method based on adaptor	It is suitable for the study of biological function, biomarker, clinical diagnosis and prognosis of exosomes	The use of adapters is limited by the limited number of adapters

Different exosome isolation methods may result in variations in concentration, purity, and activity, potentially affecting selected miRNAs or altering exosome miRNA profiles.^[21–24]^ Each method has its strengths and weaknesses, making it crucial to choose the right approach based on the specific requirements of the study or application. As research in exosome biology and diagnostics progresses, advancements in isolation techniques will likely continue, offering more efficient and reliable ways to extract and analyze exosomes. This evolution will be pivotal in unlocking the full potential of exosomes in various fields, including diagnostics, therapeutics, and biomarker discovery.

## 4. Application of exosome miRNA in prenatal diagnosis

**Figure 1.**
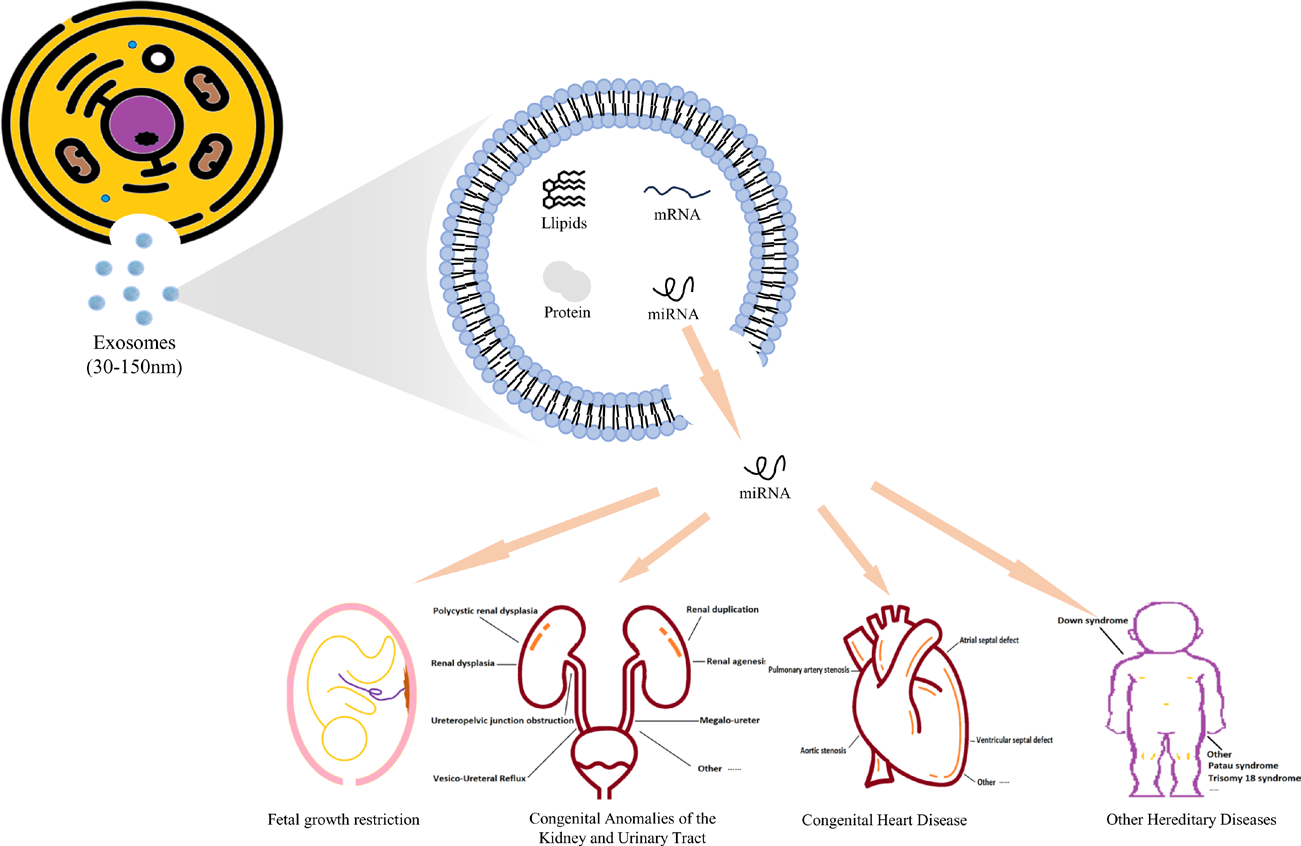
Progression and prospects in studies on exosomal miRNA for prenatal diagnosis.

Studies indicate that exosomal miRNAs play substantial biological roles in various areas, including the onset and progression of human tumors, inflammatory responses, immune responses, and angiogenesis. Exosomal miRNAs can serve as valuable biomarkers for the diagnosis and prognosis of these diseases. However, there is currently limited research on the potential applications of exosome miRNAs in the field of prenatal diagnosis. Hence, more experiments and discoveries are necessary for further exploration. (Figure 1 illustrates these results.)

### 4.1. Exosome miRNA and fetal growth restriction

Fetal growth restriction (FGR) is recognized as a severe perinatal complication, affecting between 3% and 10% of pregnancies globally.^[25]^ In China, FGR has emerged as a substantial contributor to birth defects.^[26]^ FGR not only impedes postnatal fetal development in utero, but also affects children and adolescents, manifesting as neurodevelopmental retardation, cognitive deficits, intellectual disability, and delayed physical growth. This significantly impacts the quality of life of both families and their children, augmenting the economic and social burdens on families.

During the early stage of pregnancy, the placental tissue expresses a miRNA cluster known as C14MC, comprising 52 genes including miR-379-3p, miR-369-5p, and miR-380-3p, crucial in processes like cellular growth, RNA metabolism, and transcription regulation.^[27]^ Sun et al^[28]^ observed an increase in 162 miRNAs and decrease in 71 miRNAs in amniotic fluid of mice on gestation day 17 compared with those on gestation days 13 and 15, suggesting involvement in fetal nervous system development. Zhu et al^[29]^ demonstrated that the miR-29a family serves as a pivotal regulator in the development of skeletal muscle impairment in a growth-retarded pig model by targeting CCND1 and IGF1. Luo et al^[30]^ showed a significant decrease in miR-150 expression within tissue and functionally deranged chorionic cord blood exosomes in intrauterine growth restriction piglets, while miR-150 upregulation can considerably augment human umbilical vein endothelial cell proliferation, migration, and tubulogenesis, indicating a pro-angiogenic role. This suggests that miRNAs play a crucial regulatory role in fetal development. Mouillet et al^[31]^ conducted a study revealing a 1.84-fold increase in plasma miRNA levels in pregnant women with FGR (*P* ≤ .01), while concurrently observing a 24% decrease in placental miRNA levels in FGR cases (*P* ≤ .01). They identified a correlation between altered miRNAs expression and FGR, suggesting a potential mechanism where miRNAs regulate their target gene-related mRNAs, influencing placental tissue growth, particularly in trophoblast cells. This regulation plays a crucial a role in controlling cell differentiation, migration, invasion, and apoptosis. Makrisetal et al^[32]^ identified excessive activity in exosomal miRNAs in a case of fetal growth restriction. Guichun et al^[33]^ explored the elevated expression level of miRNA-210 in the circulation of the fetal growth restriction group, suggesting potential involvement of miRNA-210 in the pathogenesis of fetal growth restriction. Cindrova-Davies et al^[34]^ meticulously analyzed placenta-derived exosomal miRNAs expression levels in 6 FGR pregnancies, revealing a notable upregulation of miR-21 compared to normal pregnancies. Rodosthenous et al^[35]^ observed associations between extracellular miRNAs (miR-127-3p, miR-20b-5p, miR-10a-5p, miR-197-3p, miR-204-5p, and miR-483-5p) in maternal blood during the second trimester with fetal growth. These studies provide compelling evidence for the pivotal role of exosomal miRNAs in FGR development. Further research in this area may aid in establishing a non-intrusive method of fetal growth monitoring.

### 4.2. Exosomal miRNA and urinary tract malformations

Congenital anomalies of the kidney and urinary tract (CAKUT) are prevalent congenital abnormalities, accounting for 15% to 20% of all fetal systemic malformations.^[36]^ These anomalies pose critical threats to the fetus^[37]^ and are the leading cause of end-stage renal disease in pediatric and adolescent patients, often necessitating kidney transplantation or dialysis.

Prenatal detection of malformed fetuses is crucial for clinical prognosis and intervention strategies. Several studies have implicated miRNAs in the onset and progression of urinary system anomalies. For instance, Xie et al^[38]^ demonstrated that Hcy regulates miR-1929-5p expression, affecting podocyte apoptosis and chronic kidney disease progression. Mitrovic et al^[39]^ found that rare CNVs downregulate hsa-miR484, potentially influencing kidney development and function. Bantounas et al^[40]^ demonstrated, using the model of human embryonic stem cells, that suppression of the miR-199a/214 cluster resulted in glomerular and tubular malformation in renal organoid tissues. Hemker et al^[41]^ showed that the absence of hypoxia-responsive miR-210 leads to a gender-specific nephron defects. Marrone et al^[42]^ found that the absence of miR-17 to 92 in renal progenitor cells in mice leads to kidney dysplasia and chronic kidney disease. Sun et al^[43]^ identified upregulation of miR-199a-5p in autosomal dominant polycystic kidney disease tissues or cells, enhancing cell proliferation by suppressing *CDKN1C*. Liu et al^[44]^ ascertained that miR-25-3p modulates the expression of ATG14-Beclin 1, affecting renal cell proliferation and autophagy in polycystic kidney mice. de Stephanis et al^[45]^ demonstrated that miR-501-5p activates the mTOR/MDM2 pathway in ADPKD cells, leading to p53 protease complex degradation. Lee et al^[46]^ examined the modulation of miR-15a on the expression of the cell cycle modulator Cdc25A and its influence on hepatic cystogenesis in an apolipoprotein E knockout mice model of polycystic kidney disease.

In patients with the urinary system disorders, Magayr et al^[47]^ found significantly reductions in miR-192-5p, miR-194-5p, miR-30a-5p, miR-30d-5p, and miR-30e-5p in renal tissues and urine exosomes. Kohl et al^[48]^ examined a collection of 96 renal growth microRNAs from 1213 individuals in 980 families with inherited congenital kidney and urinary tract disorders, discovering that mutations influencing the maturation of CAKUT individuals’ microRNAs were seldom encountered. Jovanovic et al^[49]^ identified a significant upregulation of miR-144 expression in congenital anomalies of the child’s kidneys and urinary tract.

Overall, the studies of miRNAs in CAKUT hold great promise for advancing our understanding of these conditions and developing novel therapeutic strategies. Continued research in this field could lead to significant advancements in the diagnosis, treatment, and management of CAKUT, ultimately improving the quality of life for affected individuals.

### 4.3. Exosomal miRNA and cardiovascular system malformations

In China, congenital heart disease (CHD)^[50]^ stands as the foremost birth defect, significantly impacting morbidity and mortality among newborn infants.^[51,52]^ Despite advancements in ultrasound technology and practitioner expertise, the detection rate for serious congenital malformations during pregnancy remains between 40% and 70%, with rates for less prominent abnormalities even lower.^[53,54]^ Therefore, early screening and diagnosis of fetal cardiovascular system anomalies are crucial, emphasizing the need for timely intervention.

The embryonic development of the heart is intricately regulated by numerous genes and signaling pathways, posing challenges for research into CHD pathogenesis.^[55]^ Emerging evidence suggests that non-coding RNA play a role in regulating gene expression and may contribute to heart development.^[56,57]^ Xiaochuan et al^[58]^ demonstrated the involvement of the miR-125b/RASSF1 axis in cardiac myocyte apoptosis, with miR-125b directly regulating RASSF expression. Coppola et al^[59]^ elucidate that the role of miR-99a/let-7c cluster in myocardial development through altering epigenetic factors, where let-7c augments myocardial development by upregulating smooth muscle cell specific genes (T/Bra and Nodal), as well as genes associated with cardiac development (Mesp1, Nkx2.5, and Tbx5). Shi et al^[60]^ showed maternal exosomes in diabetes may induce cardiac developmental defects via miRNA. Wang et al^[61]^ studied the role of exosomes in diabetes-related cardiovascular damage and identified Hsp20 as a key protein involved in mitigating cell disarrangement. Maimait et al^[62]^ study revealed that BMSC-Exos carrying miR-122a could alleviate diabetic cardiomyopathy symptoms by restoring cardiomyocyte autophagy levels and diminishing inflammation and cell damage.

Yu^[63]^ showed that the evaluating of miRNAs and circRNAs in maternal plasma can substantially enhance the efficacy of fetal cardiac CHD screening. They found that hsa_circ_0000992 is highly expressed in cardiovascular tissues of CHD fetuses, acting as a “molecular sieve” for hsa-miR-378g, which inhibits cardiomyocyte proliferation and migration. Hsa-miR-378g displays reduced expression in CHD fetal heart tissues, and MEIS1 which was targeted by hsa-miR-378g is strikingly overexpressed. The regulatory axis managing hsa_circ_0000992/hsa-miR-378g/MEIS1 plays an indispensable role in the progression of heart development. Significantly, Yuxia et al^[64]^ found lower expression of miR-103a-3p in peripheral blood among mothers of tetralogy of Fallot patients. Junqing et al^[65]^ showed elevated maternal serum miR-29c expression in fetal cardiac malformations, indicating potential diagnostic markers for CHD.

In summary, these research findings underscore the importance of exosomes in maintaining normal heart development and function. Further research will contribute to a better understanding of the role of exosomes in the pathogenesis of heart diseases and provide new insights for the development of novel diagnostic and therapeutic approaches.

### 4.4. Exosomes miRNA and hereditary diseases

A significant classification of diseases that attribute genetic factors, either exclusively or predominantly, is referred to as inherited disorders, primarily manifesting as abnormalities in chromosomal numerical or structural variations and alterations in molecular genes. Among these, Down syndrome (DS) stands as the most prevalent chromosome disorder in prenatal birth defect control, with an incidence rate of approximately 1/600 to 1/800. Its clinical manifestations are distinct, including delayed intellectual development, distinctive facial features, stunted growth, and multiple deformities. Therapeutic approaches for chromosomal disorders like DS are currently lacking. Thus, the implementation of antepartum diagnostic initiatives is crucial to preventing the birth of children with severe genetic diseases, intellectual impairments, and congenital deformities.

MiRNAs, widely existing, serve as an influential regulatory mechanism in gene expression, playing pivotal roles in various processes such as lymphocyte proliferation, differentiation, activation, and apoptosis. Keck-Wherley et al^[66]^ conducted a differential expression analysis of miRNAs in the hippocampal region and blood of Ts65Dn mice, identifying miR-15 as notable upregulated, potential involved in DS-related synaptic plasticity, neurogenesis impairment, and hematopoietic disorders. Shaham et al^[67]^ reported aberrant expression of miR-486-5p regulated by the GATA1 gene in DS patients with myeloid leukemia, implicated in fostering survival of abnormal red blood cell phenotypes. Chaves et al^[68]^ demonstrated that elevated expression of DYRK1A mRNA in the hippocampus of 5-month-old DS mice, concurrently associated with reduced miR-199b levels. Shi^[69]^ discovered increased miR-138-5p expression and decreased EZH2 expression in the DS hippocampus, potentially implicating neurological impairments of DS patients. Izzo et al^[70]^ discovered overexpression of miR-99a-5p, miR-155-5p, and let-7c-5p in DS infants’ fetal hearts, associated with mitochondrial dysfunction and increased risk of congenital heart disease (CHD). Farroni et al^[71]^ suggested dysregulation of miR-155 and miR-125b might be linked to compromised B cell responses in DS. Xu et al^[72]^ identified 114 miRNAs with significantly different expression patterns in the DS children’s lymphocytes. potentially involved in the immune deficiency mechanisms observed in DS. International research^[73–76]^ asserts overexpression of five miRNAs (miR-99a, let-7c, miR-125b-2, miR-155, and miR-802), located on chromosome 21, is linked to a spectrum of clinical characteristics in DS patients, including mental deficiency, childhood leukemia, immunodeficiency, and hypotension. The above study anticipates potential difference in miRNA expression in DS fetuses.

A series of studies have directly confirmed the differential miRNA expression in DS patients. Hromadníková et al^[77]^ was the first to identify 21 trisomy-derived miRNAs in maternal peripheral blood; however, no expression differences were noted between pregnant women carrying a DS fetus and those carrying a normal fetus. The reason for this might due to the elevated miRNA background in the maternal peripheral blood. In a commendable study, Xu et al^[78]^ isolated mononuclear cells from umbilical cord blood to investigate miRNA expression in fetuses with DS. A total of 149 known and 2 uncharacterized miRNAs with differing expression levels were identified, the majority of which target mRNA genes involved in immune regulation (SOD1, MXD4, PBX1, BCLAF1, and FOXO1). This interesting observation suggests that miRNA dysregulation in DS fetuses may contribute to hematopoietic abnormalities and immunodeficiency. Wuxian et al^[79]^ demonstrated that the expression of miR-nov21 is significantly elevated in mononuclear cells from the umbilical cord blood of DS fetuses compared to normal fetuses. Furthermore, miR-nov21 is involved in the regulation of cardiac and neuronal development, thus exerting significant impact on the intellectual development of DS individuals.^[80]^ Balci et al^[81]^ discovered elevated levels of hsa-miR-4732-5p and hsa-miR-181a-5p in maternal plasma during DS pregnancies. Karaca et al^[82]^ performed research on the miRNAs in amniotic fluid exosomes obtained from pregnant women with Down syndrome fetuses, discovering that miRNAs-125b-2, miRNA-155, and miRNA-3156 associated with chromosome 21 were notably elevated in the amniotic fluid of pregnant women carrying Down syndrome fetuses when compared to normal pregnancy or non-pregnant women.

In summary, studies have identified specific miRNAs with altered expression patterns in DS, providing insights into the molecular mechanisms underlying the syndrome’s pathogenesis. Further research in this area could lead to the development of novel diagnostic and therapeutic approaches for DS and other chromosomal disorders.

## 5. Summary and outlook

Current research on fetal exosomes primarily focuses on the analyzing exosomal miRNA expression profiles, but studies have shown low overlap. Factors such as inconsistent sampling criteria, diverse extraction techniques, and scarcity of samples may contribute to this disparity. However, existing studies have elucidated the vital role of exosomal miRNAs in physiological and pathological processes related to numerous birth defects (including congenital anomalies, urogenital system malformations, cardiovascular system malformations, and genetic diseases). This suggests a promising potential for using exosomal miRNAs as prospective biomarkers for prenatal genetic disease diagnosis. Nevertheless, further investigation into their specific mechanisms is warranted to pave the way for noninvasive prenatal diagnostic indicators.

## Author contributions

**Conceptualization:** Keqin Jin, Min Hu.

**Funding acquisition:** Keqin Jin,Shuangshuang Shen, Ruyong Shi, Xiayuan Xu, Min Hu.

**Resources:** Shuangshuang Shen, Ruyong Shi, Xiayuan Xu.

**Writing – original draft:** Keqin Jin.

**Writing – review & editing:** Keqin Jin, Xiayuan Xu, Min Hu.
